# Percutaneous Transretropubic Needle Prostatic Biopsy Under US and CT Guidance

**DOI:** 10.1002/iju5.70056

**Published:** 2025-05-28

**Authors:** Maki Hirao, Hideki Ishimaru, Satomi Yoshimi, Takamasa Nishimura, Taiga Oka, Chika Somagawa, Tomoki Nakano, Ryoichi Imamura, Ryo Toya

**Affiliations:** ^1^ Department of Radiology Nagasaki University Hospital Nagasaki Japan; ^2^ Departments of Radiological Sciences Nagasaki University Graduate School of Biomedical Sciences Nagasaki Japan; ^3^ Department of Urology Nagasaki University Hospital Nagasaki Japan

**Keywords:** computed tomography, percutaneous, prostate biopsy, transretropubic, ultrasound

## Abstract

**Introduction:**

No previous instances of percutaneous transretropubic prostate biopsy have been documented.

**Case Presentation:**

A 74‐year‐old male patient with a permanent stoma, who had undergone colectomy for descending colon cancer two decades earlier, reported experiencing dysuria. A screening examination revealed an elevated prostate‐specific antigen level of 120.24 ng/mL. Despite an intact rectum, the patient's anus was severely constricted or blocked, preventing both digital rectal examination and the insertion of a transrectal ultrasound probe. A transabdominal ultrasound‐guided transretropubic prostate biopsy was conducted while monitoring the needle tip position using computed tomography. The subsequent pathological analysis confirmed prostatic adenocarcinoma.

**Conclusion:**

This case represents the first reported instance of a percutaneous transretropubic prostate biopsy.

**Summary**

- For men who face challenges with transrectal ultrasound, percutaneous transretropubic prostate biopsy may serve as an alternative method for diagnosing prostate cancer.

**Abbreviations**
CT: computed tomography
US: ultrasound

## 1 Introduction

Prostate biopsy is essential for diagnosing prostate cancer, and transrectal or transperineal needle biopsies, both of which are guided by transrectal US, are commonly performed [1, 2]. However, in men with anal closure or severe anal stenosis, transrectal US is not feasible, and other alternatives are required.

We performed transabdominal US‐guided transretropubic prostate needle biopsy with the assistance of CT in a patient whose severe anal stenosis prevented the use of a transrectal US probe.

## 2 Case Report

**FIGURE 1:**
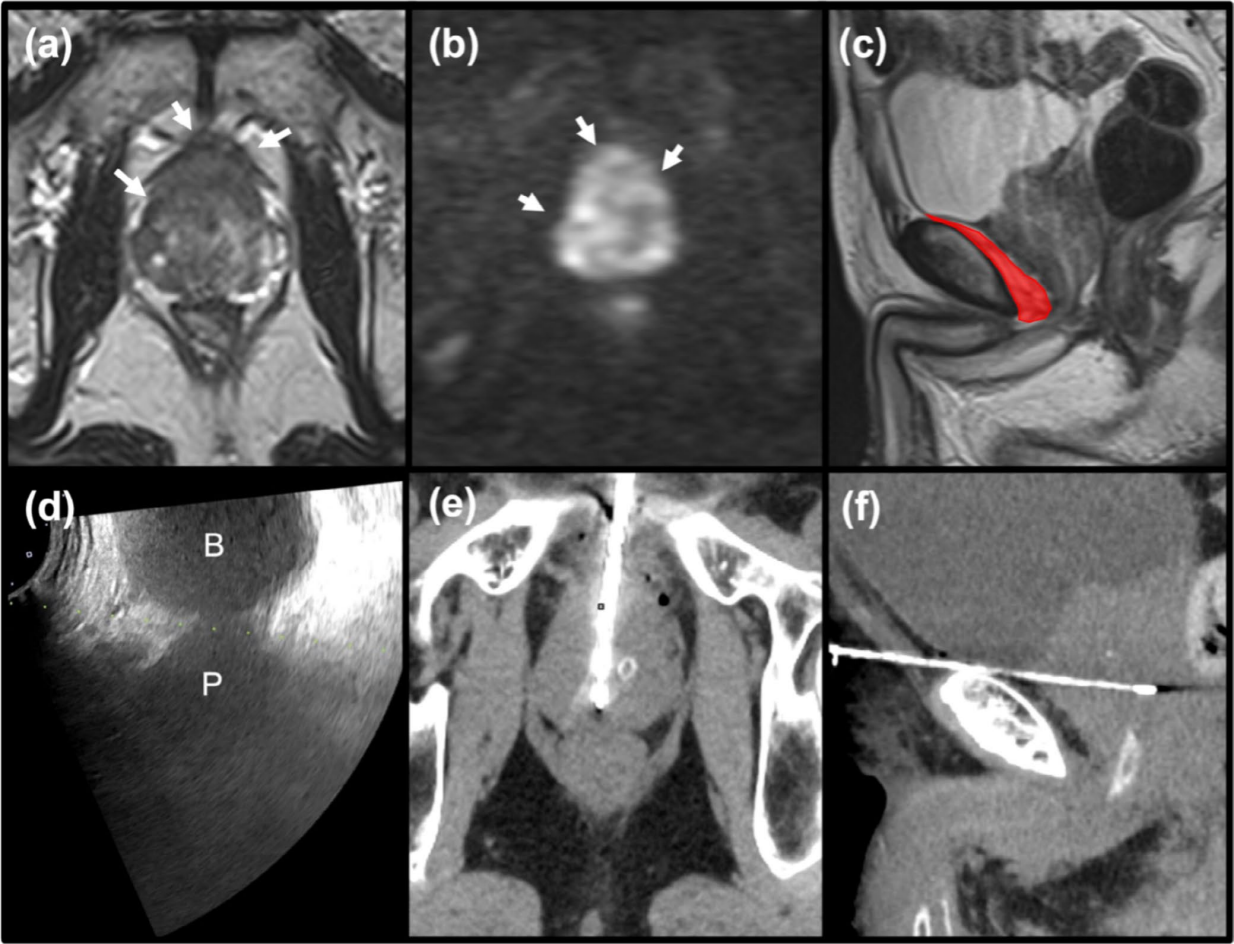
(a, b) Axial T2‐weighted image shows homogeneous hypointensity and diffusion weighted image shows hyperintensity in the anterior zone of the prostate, suggesting prostate cancer (arrows). (c) Sagittal T2‐weighted image shows the retropubic space (red). (d) Transabdominal US showing the bladder (B) and prostate (P). The needle is advanced along the virtual puncture line (dashed line). (e, f) CT reconstructed in the oblique axial and oblique sagittal planes parallel to the needle when the biopsy needle reached the prostate shows that the biopsy needle reached the right side of the prostate, avoiding the urethral catheter.

A 74‐year‐old man with a permanent stoma, who had undergone colectomy for descending colon cancer 20 years ago, presented with dysuria and was found to have a prostate‐specific antigen level of 120.24 ng/mL in screening tests. Magnetic resonance imaging revealed the anterior zone of the prostate was hypointense on T2‐weighted images and hyperintense on diffusion‐weighted images, suggesting prostate cancer (Figure 1a,b). Although the rectum remained intact, the anus was severely stenotic or obstructed, making even digital rectal examination impossible. Urologists were unable to insert a transrectal US probe and lacked experience with transperineal US‐guided biopsies; therefore, percutaneous prostate biopsy was deferred to interventional radiology.

Percutaneous CT‐guided transgluteal biopsy was considered an option; however, it was presumed that transretropubic access (Figure 1c) might be easier than transgluteal access for sampling the anterior zone of the prostate.

The procedure was performed under local anesthesia using a hybrid interventional radiography/computed tomography system. The patient was placed in the supine position with a urethral catheter inserted, and 500 mL of saline was injected into the bladder via a urethral balloon catheter to distend the bladder.

A 22‐gauge, 89‐mm spinal needle (Top spinal needle; TOP, Tokyo, Japan) was threaded into a 17‐gauge, 7‐cm coaxial needle (Co‐Axial Introducer Needle; Argon Medical Devices, The Netherlands). A microconvex‐type US transducer was placed above the pubic bone and tilted caudally. Under US guidance, the 22‐gauge spinal needle was advanced into the space between the pubic bone and bladder (retropubic space) (Figure 1d). The needle passage and anterior surface of the prostate were anesthetized with 10 mL of 1% lidocaine. Subsequently, the 17‐gauge coaxial needle was inserted coaxially through the 22‐gauge spinal needle. When the coaxial needle was inserted at the deepest point, the 22‐gauge spinal needle was removed, and the 17‐gauge guiding needle was tilted slightly to the right to prevent the biopsy needle from pointing into the urethral catheter. An 18‐gauge semiautomatic biopsy needle was directed towards the prostate through a 17‐gauge guiding needle, utilizing the urethral catheter as a reference to prevent any injury to the urethra.

CT was performed to confirm the target of the biopsy needle (Figure 1e,f). Samples were collected from two different locations in the anterior zone. The postprocedural course was uneventful, and the final pathological result was adenocarcinoma of the prostate (Gleason score 4 + 5 = 9/Group 5). The patient started androgen deprivation therapy, which is currently ongoing.

## 3 Discussion

The best predictor of tumor aggressiveness is the Gleason score, which can be obtained only by histopathological analysis of biopsy samples [3]. Thus, transrectal US biopsy of the prostate remains an essential component of diagnostic workup [1, 4]. However, when transrectal US is not possible, such as after proctectomy, alternative methods should be considered. When transrectal US is unfeasible, transperineal US‐guided prostate biopsy serves as an alternative [5]; however, this technique remains uncommon, and many urologists lack experience with it.

Strictures of the anal canal and anorectal junction most commonly occur following hemorrhoidectomy, perineal trauma, anorectal sepsis, anal fistula surgery, malignancy, or radiotherapy [6]. Although the precise reason for the patient's severe anal stenosis remains unclear, the prolonged 20‐year period of complete anal inactivity following colostomy may have affected the function of the anal region.

Papanicolau et al. reported the transgluteal biopsy of the prostate under CT guidance in men who had undergone proctocolectomy [7]. Because the transgluteal access route to the anterior zone of the prostate is long, it is likely that multiple corrections of the needle path will be necessary, and complications such as bleeding may occur. Furthermore, samples should be collected by puncturing both gluteal muscles to ensure representation from each side. Because the gluteal muscle is thick, a large resistance to the passage of the semi‐automatic biopsy needle is expected.

An important advantage of the transretropubic approach is that a single guiding needle puncture facilitates multiple biopsy needle punctures to obtain multiple specimens. Because the retropubic space is areolar tissue, blunt dissection of the retropubic space using a guiding needle is not difficult. The angles of the guiding and biopsy needles can also be changed from side to side to avoid urethral damage. The ease of adjusting the angle of the guiding and biopsy needles eliminates the need for repeated punctures to collect multiple specimens. This procedure can be performed at any facility, with the ability to combine US and CT to image the needle and its entire path. Anatomically, the transretropubic approach is advantageous for biopsies in the anterior prostate zone, although it can also be used for peripheral zone biopsies (Table 1).

**TABLE 1:** Access routes for prostate biopsy in patients underwent abdominoperineal resection and their advantages and disadvantages.

Access route	Advantages	Disadvantages
Transperineal	‐ Cost‐effective, fast approach	‐ Image quality of transperineal US is not so good ‐ Requires multiple punctures to obtain multiple specimens
Transretropubic	‐ Allows biopsy of both sides of the prostate with a single guide needle puncture ‐ Less resistance when puncturing with the biopsy needle	‐ Requires CT‐guidance, expensive, and time‐consuming's venous plexus? ‐ Risk of bleeding from Santorini‘s venous plexus?
Transgluteal	‐ No critical structures in the puncture path	‐ Requires CT‐guidance, expensive, and time‐consuming ‐ Requires puncturing both gluteal muscles for bilateral biopsy ‐ Greater resistance when puncturing with the biopsy needle

To the best of our knowledge, there have been no previous reports of transabdominal US‐guided needle biopsy of the prostate. Some previous reports have not recommended transabdominal US‐guided needle biopsy of the prostate [7, 8]. However, when using a microconvex‐type US transducer, as in the present case, inserting a needle into the retropubic space does not seem challenging.

When performing prostatectomy via the retropubic space, it is important to control the bleeding from the Santorini's venous plexus, which runs in front of the prostate. When the retropubic space is open for prostatectomy, this damage may cause massive bleeding [9]. However, no bleeding was observed in the present case, and we speculate that the needle biopsy itself did not cause massive bleeding because the retropubic space was not open. As this is a single case report, further case studies are required to determine the safety of this technique.

One shortcoming of this report is the absence of an attempt to maneuver the biopsy needle in the craniocaudal direction of the prostate. The safety of moving the needle in various directions to achieve a precise targeted biopsy remains uncertain.

## 4 Conclusion

Transabdominal US‐ and CT‐guided transretropubic prostate biopsy may be an alternative in men with difficulties in transrectal US, especially when anterior zone prostate cancer is suspected.

## Consent

Informed consent was obtained from the subject.

## Conflicts of Interest

The authors declare no conflicts of interest.
